# A multiplex marker set for microsatellite typing and sexing of sooty terns *Onychoprion fuscatus*

**DOI:** 10.1186/s13104-017-3084-9

**Published:** 2017-12-20

**Authors:** Lucy J. H. Garrett, Deborah A. Dawson, Gavin J. Horsburgh, Silas James Reynolds

**Affiliations:** 1 Centre for Ornithology, School of Biosciences, College of Life & Environmental Sciences, University of Birmingham, Edgbaston, Birmingham, B15 2TT UK; 2 Department of Animal and Plant Sciences, University of Sheffield, Sheffield, S10 2TN UK; 3 Army Ornithological Society (AOS), c/o Prince Consort Library, Knollys Road, Aldershot, Hampshire GU11 1PS UK

**Keywords:** Ascension island, Colonial seabird, Conservation, Microsatellite loci, PCR, Population genetics, Relatedness, Sex-typing

## Abstract

**Objectives:**

Seabirds have suffered dramatic population declines in recent decades with one such species being the sooty tern *Onychoprion fuscatus*. An urgent call to re-assess their conservation status has been made given that some populations, such as the one on Ascension Island, South Atlantic, have declined by over 80% in three generations. Little is known about their population genetics, which would aid conservation management through understanding ecological processes and vulnerability to environmental change. We developed a multiplex microsatellite marker set for sooty terns including sex-typing markers to assist population genetics studies.

**Results:**

Fifty microsatellite loci were isolated and tested in 23 individuals from Ascension Island. Thirty-one were polymorphic and displayed between 4 and 20 alleles. Three loci were Z-linked and two autosomal loci deviated from Hardy–Weinberg equilibrium. The remaining 26 autosomal loci together with three sex-typing makers were optimised in seven polymerase chain reaction plexes. These 26 highly polymorphic markers will be useful for understanding genetic structure of the Ascension Island population and the species as a whole. Combining these with recently developed microsatellite markers isolated from Indian Ocean birds will allow for assessment of global population structure and genetic diversity.

**Electronic supplementary material:**

The online version of this article (10.1186/s13104-017-3084-9) contains supplementary material, which is available to authorized users.

## Introduction

Sooty terns *Onychoprion fuscatus* are long-lived pelagic seabirds distributed throughout the tropical oceans where their range covers in excess of 20,000 km^2^ [1]. They are obligate colonial breeders nesting in large numbers, with birds breeding on Ascension Island in the South Atlantic Ocean constituting 40% of the Atlantic population. The long-term study of demographics and life history [2, 3], make it an ideal study population in which to investigate genetic structure and diversity. Genetic diversity is fundamental for populations to adapt to environmental change [4]. Declining and small populations often suffer from inbreeding depression and reduced genetic diversity making them vulnerable to extinction [4].

The sooty tern population on Ascension Island declined by 84% between 1942 and 2005 [3]. A recent study of long-term population trends on Ascension Island prompted an urgent call for reclassification of their IUCN (International Union for Conservation of Nature) status from ‘Least Concern’ to ‘Critically Endangered’ [3]. This decline mirrors that of seabirds globally with pelagic seabirds being the most threatened [5]. A number of issues are thought to exert pressure on seabird populations including declining fish stocks, climate change, pollution and introduced predators at breeding grounds [6].

**Fig. 1:**
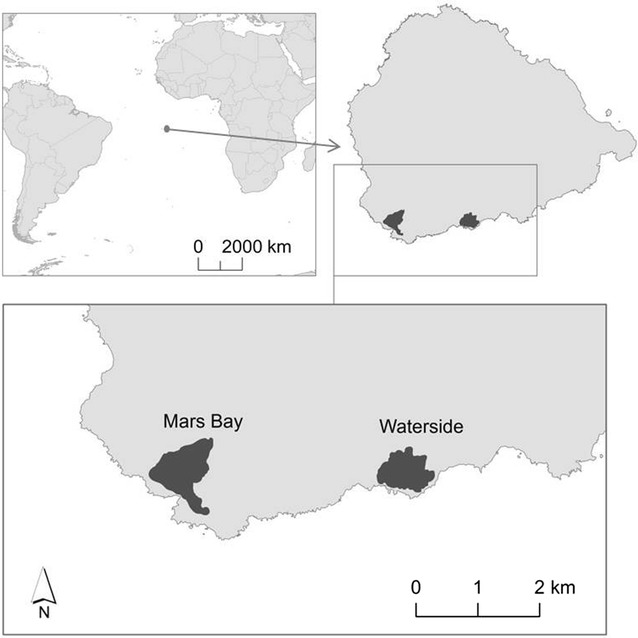
Relative locations of Ascension Island in the South Atlantic and of the study colonies of sooty terns *Onychoprion fuscatus* on the island. Dark grey areas represent the cumulative extent of peak breeding occupancy by birds between 1996 and 2016 (inclusive). Ascension Island base map source: Ascension Island Government Conservation and Fisheries Department (AIGCFD)

Since 2000 the breeding population on Ascension Island has occupied two main areas at Mars Bay and Waterside that are approx. 3 km apart (Fig. 1). Fine-scale DNA analyses would enable assessment of within-population genetic structuring and highlight potential barriers to gene flow. Microsatellite markers are an ideal tool with which to study demographic processes such as relatedness, inbreeding and genetic mixing mechanisms. Despite their widespread distribution, such processes have not been investigated in detail in this species. Sixteen microsatellite loci, were recently isolated from the Indian Ocean population [7], and given genetic differences have been documented between oceanic basins, combining these with markers derived from Atlantic Ocean birds would aid global-scale assessment of population structure. This would assist conservation management through understanding population ecology, evolutionary processes and vulnerability to environmental change.

## Main text

### Methods and Results

Genomic DNA was extracted from blood samples using an ammonium acetate precipitation method [8, 9]. Microsatellite sequences were isolated from a single adult sooty tern (BTO ring number DE97026) from a blood sample collected on Ascension Island in May 2014 at Mars Bay (Fig. 1). Sooty terns are sexually monomorphic [10], but genetic sex-typing of this individual using three sex-typing markers revealed it was a male (sex markers: Z002A, Z002D, [11] and Z43B, [12]). DNA concentration was quantified using a fluorimeter (FluostarOptima, BMG LABTECH Ltd., Aylesbury, UK) and its quality assessed by electrophoresis. The library was created by digesting the DNA with the restriction enzyme MboI and enriching the MboI fragments for the dinucleotide microsatellite motifs (CA)n, (GA)n, (TC)n,(TG)n using magnetic beads in the hybridisation [13, 14]. An Illumina paired-end library was created using 1 µg of the repeat-enriched genomic DNA. The NEBNext Ultra DNA Library Prep Kit for Illumina (New England Biolabs Ltd. Hitchin, UK) protocol was followed and sequencing conducted using a MiSeq Benchtop Sequencer (Illumina Inc., San Diego, CA, USA). Sequencing was undertaken as two × 250 bp paired-end reads generating 1,292,242 sequences. These were trimmed for quality and Illumina adapters removed using Trimmomatic [15] and paired reads aligned using FLASH [16]. SAULA/B-linker sequences [13] were removed and consensus sequences created with QDD [17]. Finally suitable microsatellites were identified using MISA [18]. A total of 423 unique sequences were selected for potential primer design, based on a minimum of five tandem repeats and a flank of 20 bp on either side of the repeat region. These displayed five to 29 uninterrupted pure repeats (di, tri, tetra, pent and hex nucleotides) or possessed compound repeat regions (37 sequences). The lengths of the sequences obtained ranged from 196 to 457 bp in length. From the 423 sequences, those with at least eight tandem repeats were selected for primer design; primer sets were designed using PRIMER3 v 0.4.0 [19, 20]. Specifications for primer selection were set at a length of 16 to 36 base pairs (optimum 20 bp), an optimal primer melting temperature of 59–61 °C (optimum 60 °C), a maximum poly-X of three tandemly repeating nucleotides e.g. AAA, presence of a G/C clamp and the default settings for all other parameters. Fifty primer sets were designed. The 5′ end of the forward primers was fluorescently labelled initially with HEX or 6-FAM. Uniqueness of each set of sequence primers was verified using BLAST software [21].

Genotyping was carried out using DNA extracted from blood samples from 23 breeding birds from Mars Bay and Waterside (12 and 11 individuals, respectively) on Ascension Island (Fig. 1) during the December 2015 breeding period. Initially, each locus was amplified separately (singleplexed) in all individuals. All loci were PCR amplified using fluorescently labelled forward primers using either 6-FAM or HEX initially (Additional file 1: Table S1). QIAGEN’s Multiplex PCR kit (QIAGEN, Manchester, UK) was used to perform PCRs following the manufacturer’s protocol, but using a 2 µl reaction volume added to 1 µl of air-dried DNA (following [22]). The same PCR profile was used to amplify each locus as follows: 95 °C for 15 min, followed by 44 cycles of 94 °C for 30 s, 56 °C for 90 s, 72 °C for 90 s and a final step of 72 °C for 30 min. Three sex-typing markers (Z002A, Z002D [11] and Z43B [12]), were included to assign sex as little sexual dimorphism exists in sooty tern plumage [10, 23]. For genotyping, 1 μl of PCR product was diluted to a ratio of 1:80 H_2_O and 1 μl of this solution was added to 9 μl formamide and 0.2 μl of 500-ROX size standard (Applied Biosystems, Warrington, UK). An ABI 3730 DNA Analyser was used to separate PCR products and alleles were scored using GENEMAPPER v 3.7 (Applied Biosystems, Foster City, CA, USA). Of the 50 primer sets tested, 14 either did not amplify or produced a non-specific product, five loci were monomorphic and three were Z-linked (the 13 males were heterozygous or homozygous but all 10 females were homozygotes with sexes identified using genetic sex-typing markers; see Additional file 1: Table S1). Multiplexing was undertaken using the same QIAGEN Multiplex PCR Kit and profile as previously outlined (see Table 1).

**Table 1:** Multiplex sets for sooty tern (*Onychoprion fuscatus*) microsatellite genotyping including three sex-typing markers

Locus	Clone name, NCBI accession number/references	Repeat motif	Primer sequence (5′–3′)		Multiplex/fluoro-label (F)	*n*	A	Observed (expected) allele size range (bp)	H_O_	H_E_	*P*_HWE_	Est null allele freq.
Ofu1	Trn17616	CA_(17)_	F	TGTTTAAGCAGTAAAGACAAAGCCTAC	7/NED	22	12	202–227	0.96	0.89	0.76	− 0.05
	LT903723		R	GGTGCGTTTAGAGTGCTTCTTTAG				(211)				
Ofu2	Trn23851	AC_(15)_	F	GGCTGTAGCGAGCAGTTAGG	2/HEX	22	8	189–359	0.78	0.80	0.74	0.02
	LT903724		R	GAAGCTTGGGTGCAGGTG				(209)				
Ofu3	Trn25452	CA_(16)_	F	GGCTGTAGCGAGCAGTTAGG	5/6-FAM	23	10	144–166	0.78	0.80	0.43	0.01
	LT903725		R	GAAGCTTGGGTGCAGGTG				(170)				
Ofu4	Trn4256	AC_(18)_	F	CCTGTTGCCAAGAAATAAATCTTAC	5/HEX	22	13	141–175	1.00	0.89	0.29	− 0.07
	LT903726		R	TGAAGAAGCGTGGCTGTG				(150)				
Ofu5	Trn171	TG_(21)_	F	TCCCTACTTGACTTTGGAAACATC	4/6-FAM	20	12	86–131	0.95	0.91	0.81	− 0.04
	LT903727		R	TGTACAACACTGTTCCATCATGC				(103)				
Ofu6	Trn352	CA_(16)_	F	GCGTTCGGCATCAAGTTAG	7/HEX	22	9	265–281	0.86	0.83	0.90	0.05
	LT903728		R	ATCCCTGCAAAGCACACAG				(282)				
Ofu7	Trn436	TG_(19)_	F	TTGCTACAAACCTTGGTTATTGAC	4/NED	22	10	154–184	0.77	0.85	0.23	0.04
	LT903729		R	GCAACCTTAGCATTACCTAGCTG				(165)				
Ofu8	Trn640	GA_(15)_	F	GGGTTACTGCTGGTCAGAGC	1/6-FAM	23	14	272–328	0.91	0.85	0.63	− 0.06
	LT903730		R	GCTCTAGGCCAATTTCATCATC				(289)				
Ofu9	Trn643	TG_(20)_	F	CTAAGCTGAAATTCCTGAACTGG	6/6-FAM	23	14	174–206	0.96	0.92	0.40	− 0.03
	LT903731		R	CAACTACAGACATCCCACAAGC				(185)				
Ofu10	Trn16824	CTT_(26)_	F	GGAAGGAGCATTCAGTCTGC	2/NED	20	17	132–210	1.00	0.95	0.91	− 0.04
	LT903732		R	GATGCTCAGATGCTTGCTAGG				(167)				
Ofu11	Trn13992	TATC_(15)_	F	AAAGTCTGTCACACATCCAACG	1/6-FAM	22	8	155–203	0.77	0.72	0.53	− 0.05
	LT903733		R	CACGGTGCCAGTTAATAATGC				(203)				
Ofu12	Trn129	CT_(14)_	F	TTAAGCAGAAAGCCAGAGTGG	3/6-FAM	22	9	300–330	0.73	0.80	0.14	0.04
	LT903734		R	CTTAGTGTGCTTGGTAAAGACTGAAC				(314)				
Ofu13	Ofu839	TCCA_(14)_	F	GAGGCCACCCTTACACCTC	6/HEX	22	8	142–171	0.86	0.83	0.46	− 0.04
	LT903735		R	AAATGAGCTTGGCTTTACGC				(169)				
Ofu14	Ofu897	CA_(14)_	F	GATCTTTCCCAGTAGCACCTATG	4/HEX	19	7	350–365	0.79	0.82	0.63	− 0.00
	LT903736		R	CCACCTGGCTGGATAACAG				(349)				
Ofu15	Trn191	CA_(14)_	F	AAAGAGTCTCCACCTGAAGCAG	2/6-FAM	22	10	333–354	0.86	0.86	0.97	− 0.02
	LT903737		R	AGCAATATCCCTGGCAGTACC				(340)				
Ofu16	Trn484	CA_(13)_	F	TTTCCTCCTGAGACTTGCGTA	6/6-FAM	22	7	314–327	0.55	0.68	0.20	0.09
	LT903738		R	AAACCAAACTGGCATCAAATAAGT				(324)				
Ofu17	Trn715	AC_(12)_	F	CACCTTATCAAGGGCAATGG	5/NED	23	10	185–207	0.74	0.87	0.11	0.08
	LT903739		R	TTGGATGGATAAAGCAAGCTG				(194)				
Ofu18	Trn269	TC_(12)_	F	ATCCCTGTCACTCCCATGAC	1/HEX	22	5	298–306	0.64	0.76	0.25	0.08
	LT903740		R	TGCACATGGAAAGTTGCTTC				(303)				
Ofu19	Trn15	AC_(12)_	F	TTAGCCCTTTACCCAAATGC	6/6-FAM	23	8	94–116	0.74	0.72	0.95	− 0.03
	LT903741		R	ATTACGTCAGCCTCCTCCAG				(115)				
Ofu20	Trn551	TTGG_(11)_	F	CCCAGTGACTCGCTTGCT	3/HEX	22	9	216–262	0.86	0.86	0.35	− 0.00
	LT903742		R	CTGCAACAGCCTTTCAGTCA				(221)				
Ofu21	Trn121	GT_(11)_	F	GGCTTAGAAATACTGCCTTTGC	5/6-FAM	22	20	269–321	1.00	0.95	0.36	0.04
	LT903743		R	CTGCTGGTCTGTAAACCATTTATC				(278)				
Ofu22	Trn652	AC_(11)_	F	TTTGCAACAGAAACCTTATCCTG	6/NED	23	6	152–164	0.70	0.70	0.35	− 0.00
	LT903744		R	TATATTGCCTCTGGCCGTTG				(162)				
Ofu23	Trn407	CCAT_(10)_	F	CCTGCATATCCCAATATCATCC	1/HEX	20	10	142–183	0.80	0.86	0.53	0.02
	LT903745		R	GGGAGGTTCAGGTTGTAATGC				(171)				
Ofu24	Trn442	ATCT_(9)_	F	ATGCATGGAAGCTGCTAACC	3/6-FAM	22	8	148–177	0.91	0.84	0.44	− 0.05
	LT903746		R	ATCTGAGGTGGTCATCATTCTTAAC				(169)				
Ofu25	Trn126	TTTGT_(8)_	F	TAGACCAGGCTGCTCAAAGC	5/HEX	22	10	221–226	0.73	0.73	0.42	0.00
	LT903747		R	TCCACCTCACCGTACTGGAT				(239)				
Ofu26	Trn825	AAAC_(8)_	F	CCTGGGAATAAACAGGAAAGC	4/6-FAM	22	4	189–198	0.64	0.59	0.59	− 0.05
	LT903748		R	ATCAGCCAAGGTTTGACCAC				(190)				
Z002A	[11]	–	–	–	2/6-FAM	13 M	1	249 (Z)	0	–	–	–
			–	–		10F	2	249 (Z) and 252 (W)	1.00	–	–	–
Z002D	[11]	–	–	–	1/6-FAM	13 M	1	127 (Z)	0	–	–	–
			–	–		10F	2	122 (W) and 127 (Z)	1.00	–	–	–
Z43B	[12]	–	–	–	7/6-FAM	13 M	1	270 (Z)	0	–	–	–
			–	–		10F	2	266 (W) and 270 (Z)	1.00	–	–	–

*NCBI* National Center for Biotechnology Information; Primer sequence *F* forward, *R* reverse, https://www.ncbi.nlm.nih.gov/bioproject/PRJEB21955, *n* is the number of individuals tested and M is the number of males and F is the number of females identified using the sex-typing markers (Z002A, Z002B [11] and Z43B [12]), A is the number of alleles observed, H_O_ is the observed heterozygosity, H_E_ is the expected heterozygosity, P_HWE_ is the probability of deviation from Hardy–Weinberg equilibrium, Est null allele Freq. is the estimated null allele frequency. The same PCR profile was used for all multiplexes as follows: 95 °C for 15 min, followed by 44 cycles of 94 °C for 30 s, 56 °C for 90 s, 72 °C for 90 s and a final step of 72 °C for 30 min

To ensure allele frequencies were not biased by over-representation of genotypes through inclusion of related individuals [24], pairwise relatedness was estimated using ML-Relate [25], and confirmed individuals were unrelated with r < 0.16 (Mean ± SD = 0.01 ± 0.03). Observed and expected heterozygosities and predicted null allele frequencies were calculated in CERVUS v3.0.7 [26]. Departures from Hardy–Weinberg equilibrium and linkage disequilibrium were estimated using GENEPOP v 4.2 [27]. To correct for multiple tests a false discovery rate control [28] was applied to linkage disequilibrium *p* values. The probability of identity, which estimates the likelihood that two unrelated individuals selected at random from the same population will have the same genotypes, was calculated using GENALEX v 6.5 [29, 30]. Two autosomal loci (Ofu28 and Ofu42) deviated from Hardy–Weinberg equilibrium (Additional file 1: Table S1). They also showed high predicted null allele frequencies (> 10%) and, as a consequence, were not included in the final multiplex. All three Z-linked loci (Ofu27, Ofu37 and Ofu43) were polymorphic (Additional file 1: Table S1) and did not deviate from Hardy-Weinberg equilibrium when assessed only in males. Although not included in the final multiplex, the three z-linked loci may be of utility for other studies. There was no evidence of significant linkage disequilibria between pairwise combinations of loci (*p* > 0.05). The 26 autosomal microsatellite loci, together with the three sex-typing markers, were combined into seven plexes by inclusion of the fluorescent dye NED (giving three dyes in total), to create a multiplex marker set using Multiplex Manager 1.2 [31] and validated (Table 1). The number of alleles per locus of the multiplexed autosomal loci ranged from four to 20 (Table 1), with an average of 10 ± 5.6 (SD) loci. Mean (± SD) observed (*H*_O_) and expected heterozygosities (*H*_E_) were 0.82 ± 0.12 and 0.82 ± 0.09, respectively. The probability of identity for the 26 loci was 4.1 × 10^−33^.

### Conclusions

This multiplex set containing a large number of novel microsatellite loci together with the three sex-typing markers will be of great utility for fine- and large-scale population genetic structure analyses. More specifically, this multiplex set offers an effective and economical approach for investigating parentage assignment, relatedness and assisting conservation management plans for this colonial seabird. Combining this multiplex set with 16 microsatellite markers recently developed for sooty terns in the Indian Ocean [7] would allow for robust global population genetic analysis of this species, given differences in genetic variance have been documented between Atlantic and Indo-Pacific populations [32]. This is poignant given the recent urgent call for the reassessment of conservation status of this species [3]. An assessment of population and global scale genetic structure and diversity would highlight vulnerability to environmental change and persistent population declines. Where evidence for genetic mixing occurs, conservation management which focuses on larger populations in isolation may be detrimental to the long-term resilience of the species as a whole.

## Limitations

The present study was carried out independently of the study by Danckwerts et al. [7] but ongoing discussions might result in collaborative testing of multiplex sets of primers on each research group’s study populations from the Atlantic and Indian Oceans. We did not have DNA available from allied species of seabirds from Ascension Island and the utility of our multiplex set for species such as brown noddies (*Anous stolidus*) and black noddies (*A. minutus*) still needs to be assessed.

## Additional file

**Additional file 1: Table S1.** Details of the 50 microsatellite markers tested in sooty terns (*Onychoprion fuscatus*). Markers Ofu1 to Ofu26 (inclusive) were included in the final multiplex set (see Table 1). Description of data: Results of the 50 microsatellite markers tested in sooty terns (*Onychoprion fuscatus*) from Ascension Island, including details of primer sequences, observed and expected allele sizes, and the analysis outcome for each primer.

## Abbreviations

*A. minutus*: *Anous minutus*
AIG: Ascension Island Government
AOS: Army Ornithological Society
BTO: British Trust for Ornithology
DNA: deoxyribonucleic acid
*H*_E_: expected heterozygosity
*H*_O_: observed heterozygosity
HWE: Hardy–Weinberg equilibrium
IUCN: International Union for Conservation of Nature
NCBI: National Center for Biotechnology Information
*O. fuscatus*: *Onychoprion fuscatus*
PCR: polymerase chain reaction
SD: standard deviation
UK: United Kingdom
