# Correction to: A multiplex marker set for microsatellite typing and sexing of sooty terns *Onychoprion fuscatus*

**DOI:** 10.1186/s13104-018-3184-1

**Published:** 2018-01-31

**Authors:** Lucy J. H. Garrett, Deborah A. Dawson, Gavin J. Horsburgh, S. James Reynolds

**Affiliations:** 1 Centre for Ornithology, School of Biosciences, College of Life & Environmental Sciences, University of Birmingham, Edgbaston, Birmingham, B15 2TT UK; 2 Department of Animal and Plant Sciences, University of Sheffield, Sheffield, S10 2TN UK; 3 Army Ornithological Society (AOS), c/o Prince Consort Library, Knollys Road, Aldershot, Hampshire GU11 1PS UK

## Correction to: BMC Res Notes (2017) 10:756 10.1186/s13104-017-3084-9

Following publication of the original article [1], one of the authors reported that his name was listed incorrectly, and that he would like his name to appear as S. James Reynolds instead of Silas James Reynolds. The latter format would confuse citations as all his previous publications are in the former format.

The original article has been updated.
