# Supplementary material for: A multiplex marker set for microsatellite typing and sexing of sooty terns Onychoprion fuscatus
Source: BMC Res Notes. 2017 Dec 20;10:756. doi: 10.1186/s13104-017-3084-9 (PMC5738816; doi:10.1186/s13104-017-3084-9)
Supplement: Supplementary file 1 — Additional file 1: Table S1. Details of the 50 microsatellite markers tested in sooty terns (Onychoprion fuscatus). Markers Ofu1 to Ofu26 (inclusive) were included in the final multiplex set (see Table 1). Description of data: Results of the 50 microsatellite markers tested in sooty terns (Onychoprion fuscatus) from Ascension Island, including details of primer sequences, observed and expected allele sizes, and the analysis outcome for each primer. [file 13104_2017_3084_MOESM1_ESM.docx]

**Additional file 1 Details of the 50 microsatellite markers tested in sooty terns (*Onychoprion fuscatus*).** Markers Ofu1 to Ofu26 (inclusive) were included in the final multiplex set (see Table 1)

| Locus | Clone name and NCBI accession number | Primer sequence (5’- 3’) | | Repeat motif | Fluoro-Label (F) | *T_m_* | *n* | A | Observed (Expected)allele size  (bp) | Outcome |
| --- | --- | --- | --- | --- | --- | --- | --- | --- | --- | --- |
| Ofu1 | Trn17616 | F | TGTTTAAGCAGTAAAGACAAAGCCTAC | CA_(17)_ | 6-FAM | 60.78 | 22 | 12 | 202-227 | Poly. See Table 1 |
|  | LT903723 | R | GGTGCGTTTAGAGTGCTTCTTTAG |  |  | 60.56 |  |  | (211) |  |
| Ofu2 | Trn23851 | F | GGCTGTAGCGAGCAGTTAGG | AC_(15)_ | HEX | 60.18 | 22 | 8 | 189-359 | Poly. See Table 1 |
|  | LT903724 | R | GAAGCTTGGGTGCAGGTG |  |  | 60.40 |  |  | (209) |  |
| Ofu3 | Trn25452 | F | GGCTGTAGCGAGCAGTTAGG | CA_(16)_ | 6-FAM | 60.74 | 23 | 10 | 144-166 | Poly. See Table 1 |
|  | LT903725 | R | GAAGCTTGGGTGCAGGTG |  |  | 60.74 |  |  | (170) |  |
| Ofu4 | Trn4256 | F | CCTGTTGCCAAGAAATAAATCTTAC | AC_(18)_ | HEX | 59.51 | 22 | 13 | 141-175 | Poly. See Table 1 |
|  | LT903726 | R | TGAAGAAGCGTGGCTGTG |  |  | 59.68 |  |  | (150) |  |
| Ofu5 | Trn171 | F | TCCCTACTTGACTTTGGAAACATC | TG_(21)_ | 6-FAM | 60.72 | 20 | 12 | 86-131 | Poly. See Table 1 |
|  | LT903727 | R | TGTACAACACTGTTCCATCATGC |  |  | 60.86 |  |  | (103) |  |
| Ofu6 | Trn352 | F | GCGTTCGGCATCAAGTTAG | CA_(16)_ | HEX | 59.43 | 22 | 9 | 265-281 | Poly. See Table 1 |
|  | LT903728 | R | ATCCCTGCAAAGCACACAG |  |  | 59.85 |  |  | (282) |  |
| Ofu7 | Trn436 | F | TTGCTACAAACCTTGGTTATTGAC | TG_(19)_ | HEX | 59.49 | 22 | 10 | 154-184 | Poly. See Table 1 |
|  | LT903729 | R | GCAACCTTAGCATTACCTAGCTG |  |  | 59.51 |  |  | (165) |  |
| Ofu8 | Trn640 | F | GGGTTACTGCTGGTCAGAGC | GA_(15)_ | 6-FAM | 59.87 | 23 | 14 | 272-328 | Poly. See Table 1 |
|  | LT903730 | R | GCTCTAGGCCAATTTCATCATC |  |  | 60.07 |  |  | (289) |  |
| Ofu9 | Trn643 | F | CTAAGCTGAAATTCCTGAACTGG | TG_(20)_ | 6-FAM | 59.44 | 23 | 14 | 174-206 | Poly. See Table 1 |
|  | LT903731 | R | CAACTACAGACATCCCACAAGC |  |  | 59.66 |  |  | (185) |  |
| Ofu10 | Trn16824 | F | GGAAGGAGCATTCAGTCTGC | CTT_(26)_ | 6-FAM | 59.96 | 20 | 17 | 132-210 | Poly. See Table 1 |
|  | LT903732 | R | GATGCTCAGATGCTTGCTAGG |  |  | 60.13 |  |  | (167) |  |
| Ofu11 | Trn13992 | F | AAAGTCTGTCACACATCCAACG | TATC_(15)_ | 6-FAM | 60.07 | 22 | 8 | 155-203 | Poly. See Table 1 |
|  | LT903733 | R | CACGGTGCCAGTTAATAATGC |  |  | 60.39 |  |  | (203) |  |
| Ofu12 | Trn129 | F | TTAAGCAGAAAGCCAGAGTGG | CT_(14)_ | 6-FAM | 59.64 | 22 | 9 | 300-330 | Poly. See Table 1 |
|  | LT903734 | R | CTTAGTGTGCTTGGTAAAGACTGAAC |  |  | 59.83 |  |  | (314) |  |
| Ofu13 | Trn839 | F | GAGGCCACCCTTACACCTC | TCCA_(14)_ | HEX | 59.52 | 22 | 8 | 142-171 | Poly. See Table 1 |
|  | LT903735 | R | AAATGAGCTTGGCTTTACGC |  |  | 59.50 |  |  | (169) |  |
| Ofu14 | Trn897 | F | GATCTTTCCCAGTAGCACCTATG | CA_(14)_ | HEX | 59.17 | 19 | 7 | 350-365 | Poly. See Table 1 |
|  | LT903736 | R | CCACCTGGCTGGATAACAG |  |  | 59.10 |  |  | (349) |  |
| Ofu15 | Trn191 | F | AAAGAGTCTCCACCTGAAGCAG | CA_(14)_ | 6-FAM | 60.05 | 22 | 10 | 333-354 | Poly. See Table 1 |
|  | LT903737 | R | AGCAATATCCCTGGCAGTACC |  |  | 60.35 |  |  | (340) |  |
| Ofu16 | Trn484 | F | TTTCCTCCTGAGACTTGCGTA | CA_(13)_ | 6-FAM | 60.00 | 22 | 7 | 314-327 | Poly. See Table 1 |
|  | LT903738 | R | AAACCAAACTGGCATCAAATAAGT |  |  | 60.16 |  |  | (324) |  |
| Ofu17 | Trn715 | F | CACCTTATCAAGGGCAATGG | AC_(12)_ | HEX | 60.32 | 23 | 10 | 185-207 | Poly. See Table 1 |
|  | LT903739 | R | TTGGATGGATAAAGCAAGCTG |  |  | 60.22 |  |  | (194) |  |
| Ofu18 | Trn269 | F | ATCCCTGTCACTCCCATGAC | TC_(12)_ | HEX | 59.77 | 22 | 5 | 298-306 | Poly. See Table 1 |
|  | LT903740 | R | TGCACATGGAAAGTTGCTTC |  |  | 59.85 |  |  | (303) |  |
| Ofu19 | Trn15 | F | TTAGCCCTTTACCCAAATGC | AC_(12)_ | 6-FAM | 59.08 | 23 | 8 | 94-116 | Poly. See Table 1 |
|  | LT903741 | R | ATTACGTCAGCCTCCTCCAG |  |  | 59.31 |  |  | (115) |  |
| Ofu20 | Trn551 | F | CCCAGTGACTCGCTTGCT | TTGG_(11)_ | HEX | 60.14 | 22 | 9 | 216-262 | Poly. See Table 1 |
|  | LT903742 | R | CTGCAACAGCCTTTCAGTCA |  |  | 60.18 |  |  | (221) |  |
| Ofu21 | Trn121 | F | GGCTTAGAAATACTGCCTTTGC | GT_(11)_ | 6-FAM | 59.44 | 22 | 20 | 269-321 | Poly. See Table 1 |
|  | LT903743 | R | CTGCTGGTCTGTAAACCATTTATC |  |  | 59.12 |  |  | (278) |  |
| Ofu22 | Trn652 | F | TTTGCAACAGAAACCTTATCCTG | AC_(11)_ | 6-FAM | 60.15 | 23 | 6 | 152-164 | Poly. See Table 1 |
|  | LT903744 | R | TATATTGCCTCTGGCCGTTG |  |  | 60.98 |  |  | (162) |  |
| Ofu23 | Trn407 | F | CCTGCATATCCCAATATCATCC | CCAT_(10)_ | HEX | 60.38 | 20 | 10 | 142-183 | Poly. See Table 1 |
|  | LT903745 | R | GGGAGGTTCAGGTTGTAATGC |  |  | 60.74 |  |  | (171) |  |
| Ofu24 | Trn442 | F | ATGCATGGAAGCTGCTAACC | ATCT_(9)_ | 6-FAM | 60.24 | 22 | 8 | 148-177 | Poly. See Table 1 |
|  | LT903746 | R | ATCTGAGGTGGTCATCATTCTTAAC |  |  | 59.80 |  |  | (169) |  |
| Ofu25 | Trn126 | F | TAGACCAGGCTGCTCAAAGC | TTTGT_(8)_ | HEX | 60.68 | 22 | 10 | 221-226 | Poly. See Table 1 |
|  | LT903747 | R | TCCACCTCACCGTACTGGAT |  |  | 60.39 |  |  | (239) |  |
| Ofu26 | Trn825 | F | CCTGGGAATAAACAGGAAAGC | AAAC_(8)_ | 6-FAM | 59.95 | 22 | 4 | 189-198 | Poly. See Table 1 |
|  | LT903748 | R | ATCAGCCAAGGTTTGACCAC |  |  | 59.97 |  |  | (190) |  |
| Ofu27 | Trn11858 | F | AGTCACCTCAGAAACTGATTTGG | TTCC_(17)_ | HEX | 59.67 | 13M | 20 | 225-447 (238) | Poly. |
|  | LT903749 | R | CGATGAGGCAGTTAGCACAG |  |  | 59.62 | 10F | 8 | 229-325 (238) | Z – Linked |
| Ofu28 | Trn15171 | F | CAGCAGACTACTGAACACCACAG | GT_(18)_ | 6-FAM | 60.08 | 21 | 18 | 119-215 | Poly. but rejected  due to deviation from HWE (p < 0.0001) and high null  allele frequency (0.16) |
|  | LT903750 | R | TTGGAGTTGGGAGAGTTTGG |  |  | 60.02 |  |  | (140) |  |
| Ofu29 | Trn18160 | F | ACCACCAGTTATTTGCTCCTTC | CT_(15)_ | 6-FAM | 59.53 | 22 | 6 | 199-218 | Mono. |
|  | LT903751 | R | TCTCTAGGGCGCCTATACTGAC |  |  | 59.90 |  |  | (204) |  |
| Ofu30 | Trn20523 | F | CCATGTCCTCCAGAGATGCT | TG _(29)_ | 6-FAM | 60.22 | 23 | 2 | 171-203 | Unreliable |
|  | LT903752 | R | GGCACTCGCATACATTCACA |  |  | 60.70 |  |  | (235) |  |
| Ofu31 | Trn3313 | F | GCAGGGAGAAATCCTGGAG | CA_(15)_ | HEX | 59.43 | 21 | 21 | 119-187 | Unreliable |
|  | LT903753 | R | GTATTTCGGCGATGCAATG |  |  | 59.85 |  |  | (146) |  |
| Ofu32 | Trn7015 | F | TAATGTCCGCCGAGCTTC | GT_(26)_ | HEX | 59.91 | 23 | - | - | No amp. |
|  | LT903754 | R | AACGGCGACTGTCAGAGTG |  |  | 60.04 |  |  | (182) |  |
| Ofu33 | Trn7163 | F | [HEX]TTCCCTTCTGCCAGGTTTC | GT_(16)_ | HEX | 60.18 | 21 | 2 | 234-387 | Mono. |
|  | LT903755 | R | GTGTTTCATTGCCGTATGTCC |  |  | 60.25 |  |  | (200) |  |
| Ofu34 | Trn7656 | F | TATGATGCATGCACACTAATGC | CA_(22)_ | 6-FAM | 59.62 | 23 | - | - | No amp. |
|  | LT903756 | R | TACCTCCCACTCCAAAGACG |  |  | 60.10 |  |  | (149) |  |
| Ofu35 | Trn8311 | F | GAAAGTCAACCTTCCCTCCAG | AC_(16)_ | 6-FAM | 60.10 | 22 | 6 | 135-145 | Unreliable |
|  | LT903757 | R | CCTGTGCTTTGAAGGGACTC |  |  | 59.84 |  |  | (150) |  |
| Ofu36 | Trn281 | F | AATACAGCCCTGCTCGTTTG | GT_(17)_ | HEX | 60.27 | 23 | - | - | No amp. |
|  | LT903758 | R | GATCATGCCCACCGACTC |  |  | 60.01 |  |  | (123) |  |
| Ofu37 | Trn381 | F | CCATCATGGTATCTGCCTTTC | GAA_(23)_ | 6-FAM | 59.40 | 13M | 14 | 160-255 (190) | Poly. |
|  | LT903759 | R | AGCATTGGTAATCACAAAATCG |  |  | 59.01 | 10F | 8 | 160-271 (190) | Z-Linked |
| Ofu38 | Trn8242 | F | TGCAATGCCAGTAGATGGAC | AC_(34)_ | HEX | 59.68 | 22 | 9 | 118-162 | Unreliable |
|  | LT903760 | R | TGCGTTCCGTTAAGCAGAG |  |  | 60.14 |  |  | (169) |  |
| Ofu39 | Trn12673 | F | TTGGATTCGCACACCATTC | ATCTC_(20)_ | 6-FAM | 60.47 | 23 | - | - | No amp. |
|  | LT903761 | R | GTGGCACTGACCAGAGGAAG |  |  | 60.86 |  |  | (293) |  |
| Ofu40 | Trn13248 | F | CTTGATAGGCAGGGATGGAG | AAAG_(38)_ | HEX | 59.65 | 23 | - | - | No amp. |
|  | LT903762 | R | CATGATTTATCTTGGCCTCTAGC |  |  | 59.28 |  |  | (293) |  |
| Ofu41 | Trn379 | F | TGACACAAGGTCAGCAGGAG | GT_(14)_ | 6-FAM | 60.02 | 19 | 14 | 136-168 | Unreliable |
|  | LT903763 | R | AGCAGACAAGCCCCAGTTC |  |  | 60.40 |  |  | (146) |  |
| Ofu42 | Trn193 | F | TGAGTTTAATGCTATCTGCCTGTTAC | TG_(13)_ | HEX | 60.10 | 22 | 11 | 222-252 | Poly. but rejected  due to deviation from HWE (p < 0.0001) and high null  allele frequency (0.23) |
|  | LT903764 | R | TCCAGCCTCAGCCACTATTC |  |  | 60.36 |  |  | (225) |  |
| Ofu43 | Trn40 | F | ACTGGACAGCTTGTCCTTGC | TG_(13)_ | 6-FAM | 60.45 | 10M | 5 | 156-168 (212) | Poly. |
|  | LT903765 | R | CGTGGGCAGAGACGATATTC |  |  | 60.62 | 10F | 4 | 160-168 (212) | Z-Linked |
| Ofu44 | Trn655 | F | GATCAGGCAGTTGGACTAAATGA | CTATC_(13)_ | HEX | 60.50 | 22 | 10 | 300-364 | Unreliable |
|  | LT903766 | R | CTGAGCCTGGAGGTCTTCAA |  |  | 60.52 |  |  | (315) |  |
| Ofu45 | Trn106 | F | ACAAGTGAGCAGGCAGCAG | TG_(13)_ | 6-FAM | 60.35 | 23 | - | - | No amp. |
|  | LT903767 | R | TTTATGGGAGCTGCTGAACC |  |  | 60.21 |  |  | (270) |  |
| Ofu46 | Trn814 | F | GTCGGGAAGAGCTGTCTCAG | GT_(11)_ | 6-FAM | 60.14 | 23 | 8 | 292-312 | Unreliable |
|  | LT903768 | R | AACCGGCAGTCAATTTGC |  |  | 59.65 |  |  | (304) |  |
| Ofu47 | Trn685 | F | TGTACATTCAGTGTCCAGTTGCT | CTAC_(11)_ | HEX | 59.72 | 19 | 10 | 276-312 | Unreliable |
|  | LT903769 | R | GATCACACTAAACTTAAGCAGAAATGA |  |  | 59.35 |  |  | (303) |  |
| Ofu48 | Trn516 | F | ACCAGAAGTGCGGAAAGATG | GGAT_(8)_ | HEX | 60.25 | 18 | 2 | 445-461 | Mono. |
|  | LT903770 | R | GGTCCTTCAGGGAGCTACG |  |  | 59.81 |  |  | (233) |  |
| Ofu49 | Trn468 | F | TTTCTTGCTGGCAGAGGTG | TGC_(8)_ | 6-FAM | 60.12 | 21 | 2 | 226-232 | Mono. |
|  | LT903771 | R | GCGAACTCATGAACTTCAACC |  |  | 59.73 |  |  | (231) |  |
| Ofu50 | Trn841 | F | TGTGGCCATGCAAGAGATAG | GTG_(8)_ | HEX | 59.82 | 22 | 2 | 263-394 | Mono. |
|  | LT903772 | R | GATCCACTGAACCACTATGTGC |  |  | 59.48 |  |  | (263) |  |

NCBI is the National Center for Biotechnology Information: <https://www.ncbi.nlm.nih.gov/bioproject/PRJEB21955>, T_m_ is the Primer melting temperature calculated using PRIMER3 v 0.4.0 (Koressaar and Remm 2007; Untergasser et al. 2012), *n* is the number of individuals tested, A number of alleles observed Poly. = polymorphic, Loci were homozygous in all females (F) genotyped but heterozygous in some males (M), suggesting Z-linkage (individuals were sexed using three markers (Z002A and Z002D [1] and Z43B [2]), Mono. = monomorphic, No amp. is where the primer failed to amplify, Unreliable is due to any of the following: amplification of non-primer peaks, poor scorability of alleles or poor consistency of controls. The PCR programme used was: 95°C for 15 min, followed by 44 cycles of 94°C for 30 s, 56°C for 90 s, 72°C for 90 s and a final step of 72°C for 30 min.

**References**

1. Dawson DA. Genomic analysis of passerine birds using conserved microsatellite loci [PhD Thesis]: University of Sheffield, UK; 2007.

2. Dawson DA, dos Remedios N, Horsburgh GJ. A new marker based on the avian spindlin gene that is able to sex most birds, including species problematic to sex with CHD markers. Zoo Biology. 2016;35:533-45. doi:10.1002/zoo.21326.
